# Antidepressant Use and Mortality Among Patients With Hepatocellular Carcinoma

**DOI:** 10.1001/jamanetworkopen.2023.32579

**Published:** 2023-09-06

**Authors:** Kuan-Lun Huang, Yi-Lung Chen, Robert Stewart, Vincent Chin-Hung Chen

**Affiliations:** 1 Tsaotun Psychiatric Center, Ministry of Health and Welfare, Nantou, Taiwan; 2 Department of Healthcare Administration, College of Medical and Health Science, Asia University, Taichung, Taiwan; 3 Department of Psychology, College of Medical and Health Science, Asia University, Taichung, Taiwan; 4 Department of Psychological Medicine, Institute of Psychiatry, Psychology and Neuroscience, King’s College London, London, United Kingdom; 5 South London and Maudsley National Health Services Foundation Trust, London, United Kingdom; 6 Department of Psychiatry, Chiayi Chang Gung Memorial Hospital, Chiayi, Taiwan; 7 School of Medicine, Chang Gung University, Taoyuan, Taiwan

## Abstract

**Importance:**

Liver cancer, primarily hepatocellular carcinoma (HCC), is the third leading cause of cancer deaths worldwide. Although some studies have proposed that antidepressants may have apoptotic effects on cancer, no study has examined the association between antidepressant use and HCC prognosis.

**Objective:**

To investigate the association between antidepressant use and mortality risk in patients with HCC.

**Design, Setting, and Participants:**

This population-based cohort study analyzed Taiwan’s National Health Insurance Research Database, which covers 99% of Taiwan’s population and includes comprehensive medical information. Patients with a new diagnosis of HCC between 1999 and 2017 were identified. Analysis took place in June 2023.

**Main Outcomes and Measures:**

All patients with HCC were followed up until 2018 to measure overall and cancer-specific mortality. To examine whether the timing of antidepressant use influenced the association with mortality, antidepressant use was examined before and after HCC diagnosis. Cox proportional hazards regression was performed to estimate hazard ratios (HRs) and the 95% CIs for the association between antidepressant use and overall mortality and cancer-specific mortality.

**Results:**

The study cohort comprised 308 938 participants, primarily consisting of older individuals (131 991 [42.7%] were aged ≥65 years) with a higher proportion of male individuals (202 589 [65.6%]). Antidepressant use before the diagnosis of HCC was not associated with lower risks of overall mortality (adjusted HR, 1.10; 95% CI, 1.08-1.12) and cancer-specific mortality (adjusted HR, 1.06; 95% CI, 0.96-1.17). However, antidepressant use after a diagnosis of HCC was associated with a lower risk of overall mortality (adjusted HR, 0.69; 95% CI, 0.68-0.70) and cancer-specific mortality (adjusted HR, 0.63; 95% CI, 0.59-0.68). The observed associations were consistent across subgroups with different antidepressant classes and comorbidities, including hepatitis B virus or hepatitis C virus infection, liver cirrhosis, and alcohol use disorders.

**Conclusions and Relevance:**

Based on this nationwide cohort study, postdiagnosis antidepressant use may be associated with lower mortality in patients with HCC. Further randomized clinical trial evaluation should be considered.

## Key Points

**Question:**

Is the use of antidepressants associated with a lower risk of death in patients with hepatocellular carcinoma (HCC)?

**Findings:**

In this population-based cohort study of 308 938 participants with HCC, the use of antidepressants after HCC diagnosis was associated with lower overall and cancer-specific mortality. The association was consistent across subgroups with different antidepressant classes and comorbidities.

**Meaning:**

This study supports an inverse association between antidepressant use after HCC diagnosis and mortality risk in patients with HCC.

## Teaser

This cohort study investigates the association between antidepressant use and mortality risk in patients with hepatocellular carcinoma.

## Introduction

Liver cancer is the sixth most commonly diagnosed cancer and the third leading cancer-related cause of death worldwide, and among the different forms of primary liver cancers, hepatocellular carcinoma (HCC) is the most common, accounting for 75% to 90% of cases.^1^ Patients with early HCC can be treated with curative therapies including resection, transplant, and ablation, leading to an expected overall survival time of more than 6 years.^2^ However, the majority (>70%) of patients with HCC have a diagnosis at advanced stages because the symptoms of early HCC are not easily detected. Surgical intervention is not suitable for those with advanced HCC,^3^ and the median survival time at this point is only 8 to 19 months.^2,4^ There is therefore an urgent need for research into alternative anticancer therapies, and drug repurposing based on the potential anticancerous effects of existing nononcological drugs is attracting interest as an approach.

Antidepressants are commonly used drugs that have potential anticancer effects.^5^ Preclinical and epidemiological studies examining the anticancer effects of antidepressants, including tricyclic antidepressants (TCAs), selective serotonin reuptake inhibitors (SSRIs), serotonin-norepinephrine reuptake inhibitors (SNRIs), and other atypical antidepressants, have increased. Early animal studies reported that SSRI use was associated with increased liver cancer,^6^ but other studies reported conflicting findings.^7^ Promising results for TCAs and SSRIs in HCC have been reported from in vitro and in vivo animal studies^8,9,10,11,12^ and epidemiological human studies.^13,14^ Although these epidemiological studies have reported associations of antidepressants with lower risk of HCC, associations with HCC prognosis have not been evaluated.

Therefore, we conducted a national cohort study to examine the association between antidepressants and HCC prognosis. We investigated overall and cancer-specific mortality as HCC prognosis indices. To examine whether the timing of antidepressant use influenced the association with HCC prognosis, we distinguished between antidepressant use before and after HCC diagnosis.

## Methods

### Study Design and Population

Enrollment in Taiwan’s National Health Insurance program is mandatory for all residents; it is run by the Taiwanese government and covers 99% of Taiwan’s population. The National Health Insurance Research Database (NHIRD) is comprehensive and includes information on medical procedures, prescriptions, and diagnoses in outpatient, inpatient, and emergency care. Individual medical records included in the NHIRD are anonymized to protect patient privacy. Diseases in the NHIRD were diagnosed and recorded using the *International Classification of Diseases, Ninth Revision, Clinical Modification (ICD-9-CM)* before 2015 and the *International Statistical Classification of Diseases, Tenth Revision, Clinical Modification (ICD-10-CM)* after 2015. Validation studies have demonstrated the validity of NHIRD diagnosis codes. The validity of cancer diagnosis recorded in the NHIRD has been tested by comparison with the Taiwan Cancer Registry and the positive predictive value was 93% for liver cancer diagnosis recorded in NHIRD.^15^ High concordance between claim records for medication use in the NHIRD and patient self-report has also been established.^16^ The study was approved by the Research Ethics Committee of the Chang Gung Medical Foundation. Written informed consent was not needed because this study used Taiwan’s NHIRD, which covers all residents, with research legitimacy affirmed by the Supreme Administrative Court in 2017. This study was conducted in accordance with the Strengthening the Reporting of Observational Studies in Epidemiology (STROBE) reporting guideline.

### Cohort Selection

A cohort of patients with a new HCC diagnosis was identified based on *ICD-9-CM* code 155.0 and *ICD-10-CM* codes C22.0, C22.2, C22.7, and C22.8 recorded in the NHIRD between January 1, 1999, and December 31, 2017. It has been reported that use of prevalent case may result in selection bias.^17^ To exclude prevalent cases of HCC that occurred before January 1, 1999, a 1-year exclusion assessment window was applied from January 1 to December 31, 1998. By excluding prevalent cases of HCC, we identified incident cases of HCC for analysis. The cohort entry date was the date of HCC diagnosis. The end of this study was December 31, 2018, to ensure a minimum 1-year follow-up window.

### Main Exposure

Antidepressant prescription records were obtained from the NHIRD based on the Anatomical Therapeutic Chemical (ATC) code N06A. Antidepressant use was indicated by the presence of 1 or more prescriptions for antidepressants. We divided antidepressants into 3 classes: (1) SSRIs (ATC code N06AB), (2) SNRIs (ATC codes N06AX16 and N06AX21), and (3) TCAs (ATC code AN06AA). Individuals in each antidepressant subgroup were not mutually exclusive. In addition to these 3 major classes of antidepressants, the users of remaining atypical antidepressants such as bupropion, mirtazapine, and trazodone were categorized as other antidepressants in Table 1 but were not counted in the subgroup analysis due to heterogeneity. To examine whether the timing of antidepressant use influenced the association between antidepressant use and mortality, we conducted separate analyses for antidepressant use before and after HCC diagnosis. In each comparison, the groups using antidepressants before and after the diagnosis of HCC were not mutually exclusive.

**Table 1.** Characteristics of Patients With HCC by Antidepressant Use Before or After Diagnosis

Characteristic	No. (%)			
	Antidepressant use in a year before HCC diagnosis		Antidepressant use after HCC diagnosis	
	Antidepressant use (n = 21 202)	Nonuse (n = 287 736)^a^	Antidepressant use (n = 66 211)	Nonuse (n = 235 083)^b^
Age at diagnosis, y				
<45	1828 (8.6)	42 907 (14.9)	9951 (15.0)	34 332 (14.6)
45-54	3230 (15.2)	54 563 (19.0)	12 886 (19.5)	43 904 (18.7)
55-64	4820 (22.7)	69 599 (24.2)	16 391 (24.8)	56 462 (24.0)
≥65	11 324 (53.4)	120 667 (41.9)	26 983 (40.8)	100 385 (42.7)
Sex				
Male	12 593 (59.4)	189 996 (66.0)	38 361 (57.9)	159 427 (67.8)
Female	8609 (40.6)	97 740 (34.0)	27 850 (42.1)	75 656 (32.2)
Low-income status	551 (2.6)	3992 (1.4)	1428 (2.2)	3021 (1.3)
Comorbidity^c^				
Hepatitis B virus	6990 (33.0)	107 967 (37.5)	23 044 (34.8)	89 567 (38.1)
Hepatitis C virus	5326 (25.1)	51 350 (17.8)	15 662 (23.7)	39 525 (16.8)
Liver cirrhosis	12 016 (56.7)	163 219 (56.7)	32 872 (49.6)	137 815 (58.6)
Alcohol use disorder	1614 (7.6)	10 145 (3.5)	3653 (5.5)	7706 (3.3)
CCI score, median (IQR)	10 (7-14)	8 (6-12)	9 (6-13)	8 (6-12)
HCC treatment^d^				
Hepatic operation	NA	NA	13 207 (19.9)	38 297 (16.3)
RFA	NA	NA	9431 (14.2)	25 738 (10.9)
TAE/TACE	NA	NA	25 314 (38.2)	90 213 (38.4)
Radiotherapy	NA	NA	22 306 (33.7)	81 320 (34.6)
Chemotherapy	NA	NA	523 (0.8)	11 053 (4.7)
Sorafenib	NA	NA	1300 (2.0)	14 579 (6.2)
Antidepressant subclasses, No. (%)				
SSRI	5948 (28.1)	NA	23 158 (35.0)	NA
SNRI	1325 (6.2)	NA	6993 (10.6)	NA
TCA	9597 (45.3)	NA	40 949 (61.8)	NA
Other antidepressants	5702 (26.9)	NA	11 568 (17.5)	NA

Abbreviations: CCI, Charlson Comorbidity Index; HCC, hepatocellular carcinoma; NA, not applicable; RFA, radiofrequency ablation; SNRI, serotonin-norepinephrine reuptake inhibitor; SSRI, selective serotonin reuptake inhibitor; TACE, transcatheter arterial chemoembolization; TAE, transcatheter arterial embolization; TCA, tricyclic antidepressant.

^a^ Without an antidepressant prescription in the 1 year before the HCC diagnosis.

^b^ Without an antidepressant prescription in the 1 year before and after the HCC diagnosis.

^c^ Comorbidity before HCC diagnosis.

^d^ Procedures and medication use recorded after HCC diagnosis.

For comparisons of antidepressant use prior to diagnosis, antidepressant use was defined as having at least 1 antidepressant prescription in the 1-year exposure window prior to HCC diagnosis, regardless of prior antidepressant use. Nonuse was defined as individuals who had no prescriptions for antidepressants in the 1-year exposure window prior to HCC diagnosis.

For comparisons of antidepressant use after diagnosis, those taking antidepressants were defined by at least 1 antidepressant prescription in the exposure assessment window after the date of first HCC diagnosis to death or the end of 2017. Antidepressant use was considered as a time-varying exposure to avoid potential immortal time bias.^18^ Specifically, for individuals taking antidepressants, the period between HCC diagnosis and the time of first antidepressant plus a 90-day induction period^19^ was classified as nonexposure time, and the period thereafter during follow-up was classified as exposure time. To examine the influence of induction period on the results, we performed sensitivity analysis using different induction period times (0 and 180 days). Nonusers were defined as individuals who had no prescriptions for antidepressants in the 1 year before or after HCC diagnosis to death or the end of 2017. For nonusers, their entire follow-up period was classified as nonexposure time.

### Covariates

Study covariates were included based on their relevance to antidepressant use and HCC prognosis and included demographic characteristics, comorbidities, and treatments for HCC.^20,21^ Covariate assessment windows were the time before the HCC diagnosis for relevant comorbidities, at the date of HCC diagnosis for demographic characteristics, and the time after the HCC diagnosis for HCC treatments. Among these covariates, treatments for HCC were exclusive to analysis of antidepressant use after HCC diagnosis. HCC treatment variables are more likely to be mediators of prediagnostic analyses, so adjusting for such variables is unnecessary overadjustment.^22^

Comorbidities including hepatitis B virus (HBV) infection, hepatitis C virus (HCV) infection, liver cirrhosis, alcohol use disorder, and other diseases were defined as being present if data on diagnostic codes corresponding to these comorbidities were assigned to the patient for at least 3 outpatient visits or at least 1 hospital admission before HCC diagnosis. We used the Charlson Comorbidity Index (CCI) to determine general health conditions and the overall burden of comorbidities.^20,23^

Treatments for HCC after HCC diagnosis were considered as covariates in the analysis of antidepressant use after HCC diagnosis.^20^ Data regarding HCC treatments, including hepatic operation (lobectomy, segmentectomy, hepatectomy, liver transplant), radiofrequency ablation, transcatheter arterial embolization, and transcatheter arterial chemoembolization, were retrieved from NHIRD inpatient and outpatient databased on *ICD-9* and *ICD-10* procedure codes. Data regarding sorafenib and chemotherapy for HCC, including fluorouracil, gemcitabine, docetaxel, irinotecan, doxorubicin, mitomycin, cisplatin, carboplatin, and oxaliplatin, were obtained from the NHIRD using ATC codes.

### Study Outcomes

Overall mortality and cancer-specific mortality were the primary outcomes in this study and were based on cause of death records in the NHIRD. Cancer-specific deaths were identified in the primary cause of death certification records based on *ICD-9-CM* code 155.0 and *ICD-10-CM* codes C22.0, C22.2, C22.7, and C22.8. The follow-up window for mortality was from the date of HCC diagnosis to the study end point (December 31, 2018).

### Statistical Analysis

All data analysis was performed using SAS statistical software version 9.4 (SAS Institute) and was conducted on June 5, 2023. A 2-sided hypothesis test was used with a significance level set at .05.

We reported the distribution of demographics, comorbidities, and hepatic treatment and median and IQR of CCI score between the antidepressant use and nonuse groups. Cox proportional hazards regression was performed to estimate hazard ratios (HRs) with 95% CIs for associations between antidepressant use before and after HCC diagnosis and the rates of overall and cancer-specific mortality, with adjustments for demographic factors, comorbidities, and hepatic treatment. Both crude and adjusted HRs were reported to provide a comprehensive understanding of the associations. 95% CIs that did not include 1 indicate statistical significance. Dose-response analyses were conducted to examine whether the duration of antidepressant use had a differential association with overall and cancer-specific mortality rates. We categorized the duration of antidepressant use 2 groups: short-term users (1 to 90 days) and long-term users (>90 days) because the practice of providing refillable prescriptions for up to 3 months to patients with chronic conditions in Taiwan; other studies also used such duration lengths.^24,25,26^ To investigate the association of antidepressant therapy with the prognosis of HCC in different etiological subgroups, we conducted a subgroup analysis focusing on specific subgroups, including HBV infection, HCV infection, liver cirrhosis, and alcohol use disorder. To determine whether there is an additional association of antidepressant use in conjunction with chemotherapy or sorafenib, we conducted moderation analysis to examine the association of combined use of sorafenib or chemotherapy with antidepressant compared with mortality.

To address the type I error that may result from multiple comparisons and to provide more reliable results, we adjusted the *P* values for false discovery rate in both the main analysis and the subgroup analysis.^27^

## Results

### Study Cohort

**Figure 1.**
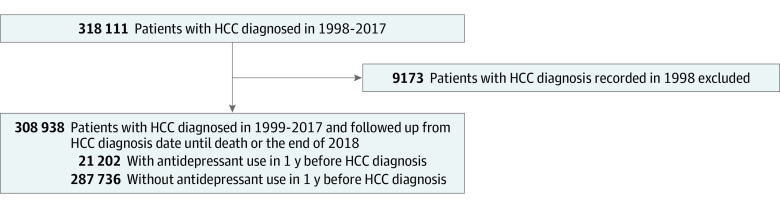
Flowchart of Study Participants Based on Antidepressant Use Before Diagnosis HCC indicates hepatocellular carcinoma.

**Figure 2.**
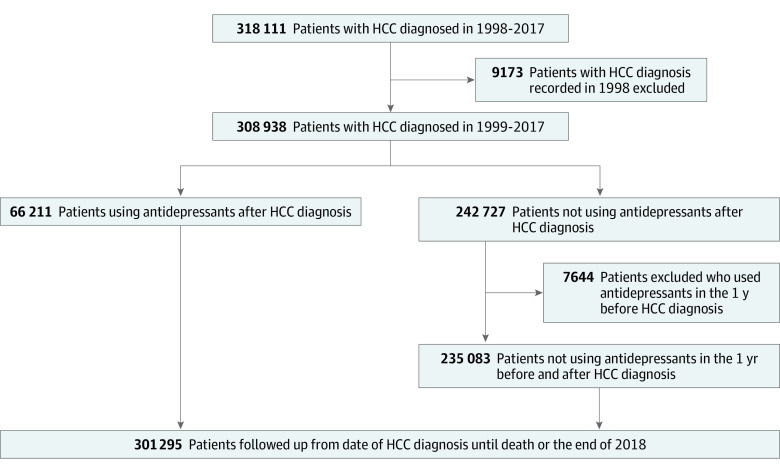
Flowchart of Study Participants Based on Antidepressant Use After Diagnosis HCC indicates hepatocellular carcinoma.

A total of 308 938 patients with HCC between 1999 and 2017 were identified from the NHIRD, after excluding 9173 patients with HCC diagnosis before January 1, 1999. For the comparison of antidepressant use before HCC diagnosis, we identified 21 202 patients with antidepressant exposure within 1 year before HCC diagnosis, and we had a nonuser group of 287 736 individuals. For the comparison of antidepressant use after HCC diagnosis, we identified 66 211 patients with antidepressant prescriptions after HCC diagnosis, and there were 235 083 nonusers in the comparison group (Figure 1 and Figure 2). The distributions of baseline characteristics for each group in the 2 comparisons are shown in Table 1. Across all groups, the majority of HCC diagnoses occurred in older individuals, with individuals 65 years and older ranging from 26 983 (40.8%) and 11 324 (53.4%) in participants based on antidepressant use before and after diagnosis, respectively. Male individuals predominated in terms of sex distribution, accounting for 38 361 participants (57.9%) and 159 427 participants (42.7%) based on antidepressant use before and after diagnosis, respectively. Individuals taking antidepressants, both before or after HCC diagnosis, were more likely to be female or have low-income status, HCV, alcohol use disorders, or higher CCI scores than nonusers. The antidepressant use group after HCC diagnosis had higher rates of undergoing hepatic operation and radiofrequency ablation and lower rates of receiving chemotherapy and sorafenib therapy than the nonuse group.

### Association Between Mortality and Antidepressant Use Before HCC Diagnosis

The crude overall mortality rates were 15.68 per 100 person-years in the antidepressant use group and 12.14 per 100 person-years in the nonuse group (Table 2). In patients with HCC, use of any type of antidepressant within 1 year before diagnosis was not associated with a lower overall mortality but instead with a slightly higher risk after adjustment for covariates (adjusted HR, 1.10; 95% CI, 1.08-1.12). Additional analyses were conducted to examine the specific association of antidepressant types, including SSRI, SNRI, and TCA, with overall mortality. The results revealed no association with reduction in overall mortality, with adjusted HRs ranging from 1.03 (95% CI, 1.00-1.05) to 1.16 (95% CI, 1.07-1.25) when compared with nonuse.

**Table 2.** Association Between Antidepressant Use and Mortality in Patients With HCC

Factor	Antidepressant use 1 y before diagnosis^a^							Antidepressant use after diagnosis^b^						
	Deaths, No.	Person-years	Mortality rate (per 100 person-years)	Crude HR (95% CI)	*P* value^c^	Adjusted HR (95% CI)	*P* value^c^	Deaths, No.	Person-years	Mortality rate (per 100 person-years)	Crude HR (95% CI)	*P* value^c^	Adjusted HR (95% CI)	*P* value^c^
**Overall mortality**														
Antidepressant use	13 616	86 864	15.68	1.09 (1.07-1.11)	<.001	1.10 (1.08-1.12)	<.001	31 908	318 122	10.03	0.73 (0.72-0.74)	<.001	0.69 (0.68-0.70)	<.001
Cumulative duration, d														
1-90	10 140	64 108	15.82	1.39 (1.36-1.42)	<.001	1.11 (1.09-1.14)	<.001	18 817	177 803	10.58	0.72 (0.70-0.73)	<.001	0.69 (0.68-0.70)	<.001
>90	3476	22 756	15.28	1.36 (1.32-1.41)	<.001	1.07 (1.04-1.11)	<.001	13 091	140 319	9.33	0.75 (0.73-0.76)	<.001	0.68 (0.66-0.69)	<.001
Subclasses														
SSRI	3475	25 496	13.63	0.98 (0.95-1.02)	.20	1.13 (1.09-1.17)	<.001	9419	209 035	4.51	0.59 (0.58-0.60)	<.001	0.62 (0.61-0.64)	<.001
SNRI	708	5205	13.60	1.04 (0.97-1.12)	.27	1.16 (1.07-1.25)	<.001	2422	66 961	3.62	0.54 (0.52-0.57)	<.001	0.60 (0.57-0.62)	<.001
TCA	8045	53 451	15.05	1.02 (1.00-1.05)	.050	1.03 (1.00-1.05)	.050	20 687	413 524	5.00	0.63 (0.62-0.64)	<.001	0.60 (0.59-0.60)	<.001
Nonuse	174 415	1 437 045	12.14	1 [Reference]	NA	1 [Reference]	NA	150 026	970 848	15.45	1 [Reference]	NA	1 [Reference]	NA
**Cancer mortality**														
Antidepressant use	455	86 864	0.52	1.00 (0.90-1.10)	>.99	1.06 (0.96-1.17)	.25	1022	318 122	0.32	0.57 (0.53-0.62)	<.001	0.63 (0.59-0.68)	<.001
Cumulative duration, d														
1-90	338	64 108	0.53	1.27 (1.14-1.42)	<.001	1.08 (0.96-1.20)	.20	618	177 803	0.35	0.59 (0.53-0.64)	<.001	0.65 (0.59-0.71)	<.001
>90	117	22 756	0.51	1.24 (1.03-1.48)	.02	1.01 (0.84-1.22)	.92	404	140 319	0.29	0.56 (0.50-0.62)	<.001	0.61 (0.54-0.67)	<.001
Subclasses														
SSRI	116	25 496	0.45	0.98 (0.95-1.02)	.20	1.13 (1.09-1.17)	<.001	286	209 035	0.14	0.42 (0.37-0.48)	<.001	0.53 (0.47-0.61)	<.001
SNRI	25	5205	0.48	0.92 (0.62-1.36)	.68	0.99 (0.67-1.47)	.96	80	66 961	0.12	0.42 (0.34-0.54)	<.001	0.54 (0.42-0.68)	<.001
TCA	274	53 451	0.51	0.98 (0.87-1.10)	.74	1.05 (0.93-1.19)	.43	622	413 524	0.15	0.47 (0.43-0.52)	<.001	0.54 (0.49-0.59)	<.001
Nonuse	6041	1 437 045	0.42	1 [Reference]	NA	1 [Reference]	NA	5286	970 848	0.54	1 [Reference]	NA	1 [Reference]	NA

Abbreviations: HCC, hepatocellular carcinoma; HR, hazard ratio; SSRI, selective serotonin reuptake inhibitor; SNRI, serotonin-norepinephrine reuptake inhibitor; TCA, tricyclic antidepressant.

^a^ Adjusted for age, sex, low income, prediagnostic comorbidities (hepatitis B virus, hepatitis C virus, liver cirrhosis, alcohol use disorder), and Charlson Comorbidity Index score. Nonuse was defined as patients with HCC without an antidepressant prescription in the 1 year before the HCC diagnosis.

^b^ Adjusted for age, sex, low income, prediagnostic comorbidities (hepatitis B virus, hepatitis C virus, liver cirrhosis, alcohol use disorder), Charlson Comorbidity Index score, and HCC treatment (operation, radiofrequency ablation, transcatheter arterial embolization/transcatheter arterial chemoembolization, radiotherapy, chemotherapy, sorafenib). Nonuse was defined as patients with HCC without an antidepressant prescription in the 1 year before and after the HCC diagnosis.

^c^ False discovery rate–adjusted *P* value. The total numbers of multiple tests adjusted in the false discovery rate method is 11 for crude and adjusted HRs.

A similar analysis was conducted for cancer-specific mortality. The crude cancer-specific mortality rate was 0.52 per 100 person-years in the antidepressant use group and 0.42 per 100 person-years in the nonuse group. We observed that antidepressant use before HCC diagnosis did not have a significant association with lower cancer-specific mortality (adjusted HR, 1.06; 95% CI, 0.96-1.17). When examining specific subgroups of antidepressants, none of them showed an association with the lower cancer-specific mortality, with adjusted HRs ranging from 0.99 (95% CI, 0.67-1.47) to 1.13 (95% CI, 1.09-1.17) when compared with nonuse.

When examining the duration of antidepressant use in the 1-year exposure window prior to HCC diagnosis, both short-term (≤90 days) and long-term (>90 days) antidepressant use displayed similar results regarding overall mortality (adjusted HR, 1.11; 95% CI, 1.09-1.14 and 1.07; 95% CI, 1.04-1.11, respectively) and cancer-specific mortality (adjusted HR, 1.08; 95% CI, 0.96-1.20 and 1.01; 95% CI, 0.84-1.22, respectively).

### Association Between Mortality and Antidepressant Use After HCC Diagnosis

The crude overall mortality rate was 10.03 per 100 person-years in the antidepressant use group after HCC diagnosis, lower than 15.45 per 100 person-years in the nonuse group. The crude overall mortality rate for antidepressant subgroups was even lower, ranging from 3.62 to 5.00 per 100 person-years. After adjustment for confounders, use of any antidepressant after diagnosis was associated with lower overall mortality (adjusted HR, 0.69; 95% CI, 0.68-0.70). When examining specific subgroups of antidepressants, the lower overall mortality were associated with postdiagnosis use of SSRI (adjusted HR, 0.62; 95% CI, 0.61-0.64), SNRI (adjusted HR, 0.60; 95% CI, 0.57-0.62), or TCA (adjusted HR, 0.60; 95% CI, 0.59-0.60).

The crude cancer-specific mortality rates were 0.32 per 100 person-years in the combined antidepressant group, 0.12 to 0.15 per 100 person-years in different antidepressant subgroups, and 0.54 per 100 person-years in nonusers. Postdiagnosis antidepressant use was associated with a lower cancer-specific mortality (adjusted HR, 0.63; 95% CI, 0.59-0.68). The antidepressant subgroups, including SSRI (adjusted HR, 0.53; 95% CI, 0.47-0.61), SNRI (adjusted HR, 0.54; 95% CI, 0.42-0.68), and TCA (adjusted HR, 0.54; 95% CI, 0.49-0.59), demonstrated an inverse association with cancer-specific mortality.

There was no apparent difference observed between short-term (≤90 days) and long-term (>90 days) antidepressant use in terms of overall mortality (adjusted HRs, 0.69 [95% CI, 0.68-0.70] and 0.68 [95% CI, 0.66-0.69], respectively) and cancer-specific mortality (adjusted HRs, 0.65 [95% CI, 0.59-0.71] and 0.61 [95% CI, 0.54-0.67], respectively). The statistical significance inferred using the false discovery rate for multiple comparisons remained the same as it did with the 95% CIs.

To examine the possible influence of the length of the induction period, we used different induction periods (0 days and 180 days) to examine the association of postdiagnosis antidepressant use, including the use of different antidepressant types, with overall and cancer-specific mortality. The results were similar to those for a 90-day induction period (eTable 1 in Supplement 1).

### Association Between Mortality and Antidepressant Use After HCC Diagnosis With Different Comorbidities

A subgroup analysis was conducted to examine the use of antidepressants after diagnosis in different comorbidity subgroups. Analyses showed significant inverse associations between postdiagnosis antidepressant use and both overall and cancer-specific mortality in patients with HCC with HBV infection (adjusted HRs, 0.76 [95% CI, 0.75-0.78] and 0.67 [95% CI, 0.60-0.75], respectively), HCV infection (adjusted HRs, 0.92 [95% CI, 0.89-0.94] and 0.86 [95% CI, 0.76-0.98], respectively), liver cirrhosis (adjusted HRs, 0.76 [95% CI, 0.75-0.77] and 0.71 [95% CI, 0.65-0.77], respectively), and alcohol use disorder (adjusted HRs, 0.76 [95% CI, 0.72-0.80] and 0.72 [95% CI, 0.53-0.98], respectively) compared with nonuse (Table 3). All the adjusted *P* values using the false discovery rate remained statistical significance (<.05).

**Table 3.** Association Between Antidepressant Use and Mortality in Different Etiological Subgroups of Patients With HCC

Subgroup	Antidepressant use after diagnosis			
	Crude HR (95% CI)	*P* value^a^	Adjusted HR (95% CI)	*P* value^b^
**HBV**				
Overall mortality	0.78 (0.77-0.80)	<.001	0.76 (0.75-0.78)	<.001
Cancer mortality	0.63 (0.57-0.70)	<.001	0.67 (0.60-0.75)	<.001
**HCV**				
Overall mortality	0.87 (0.85-0.90)	<.001	0.92 (0.89-0.94)	<.001
Cancer mortality	0.77 (0.68-0.88)	<.001	0.86 (0.76-0.98)	.02
**Liver cirrhosis**				
Overall mortality	0.76 (0.75-0.77)	<.001	0.76 (0.75-0.77)	<.001
Cancer mortality	0.65 (0.60-0.70)	<.001	0.71 (0.65-0.77)	<.001
**Alcohol use disorder**				
Overall mortality	0.65 (0.62-0.69)	<.001	0.76 (0.72-0.80)	<.001
Cancer mortality	0.52 (0.38-0.70)	<.001	0.72 (0.53-0.98)	.04

Abbreviations: HBV, hepatitis B virus; HCC, hepatocellular carcinoma; HCV, hepatitis C virus; HR, hazard ratio.

^a^ Adjusted for age, sex, low income, prediagnostic comorbidities (HBV, HCV, liver cirrhosis, alcohol use disorder), Charlson Comorbidity Index score, and HCC treatment (operation, radiofrequency ablation, transcatheter arterial embolization/transcatheter arterial chemoembolization, radiotherapy, chemotherapy, sorafenib). Nonuse was defined as patients with HCC without an antidepressant prescription in the 1 year before and after the HCC diagnosis.

^b^ False discovery rate–adjusted *P* value. The total numbers of multiple tests adjusted in the false discovery rate method is 8 for crude and adjusted HRs.

### Association Between Mortality and Antidepressant Use After HCC Diagnosis With Chemotherapy or Sorafenib

To investigate the potential effect of combination therapies, we examined the interaction between postdiagnosis antidepressant use and HCC treatments such as chemotherapy and sorafenib. None of the interaction tests between antidepressant and chemotherapy or antidepressant and sorafenib revealed additional beneficial associations with overall mortality or cancer-specific mortality. This finding was consistent across different antidepressant subgroups (eTable 2 in Supplement 1).

## Discussion

This is the first national cohort study on the association between antidepressant use and mortality risk in patients with HCC. Our results demonstrate that the use of antidepressants after HCC diagnosis, including SSRI, SNRI, and TCA, was associated with decreased both overall and cancer-specific mortality in a large, representative cohort. Notably, we observed a consistent inverse association between postdiagnosis antidepressant use and mortality risk across various comorbidity subgroups, encompassing HBV infection, HCV infection, liver cirrhosis, and alcohol use disorder. In contrast, no association was observed between the use of antidepressants before HCC diagnosis and reduced cancer-specific mortality or all-cause mortality.

Our study conducted 2 comparisons; examining the associations of antidepressant use before and after HCC diagnosis with mortality. The results showed inconsistent findings. Antidepressant use before HCC diagnosis was not associated with lower mortality risk, indicating that the antidepressant use prior to HCC diagnosis may not have an association with the severity of HCC and its subsequent prognosis. However, the use of antidepressants after HCC diagnosis was inversely associated with both overall and cancer-specific mortality. This suggests that antidepressant use might be involved in the prognosis of cancer, not before its induction. A similar finding was also observed that statin use after HCC diagnosis associated with lower HCC mortality but not in statin use before HCC diagnosis.^20^ Further studies are warranted to examine potential mechanisms of antidepressants use on HCC mortality.

Considering the inverse association between overall mortality and antidepressant use after HCC diagnosis, several noncancer causes of death, such as unintentional injury, self-inflicted injury, and suicide, have been reported in patients with cancer, which may be reduced by antidepressant use. For example, risks of motor vehicle crashes^28^ and suicide attempts have been reported to decrease following the initiation of antidepressant treatment. Furthermore, depression has been commonly observed in patients with HCC and has been associated with a lower quality of life, lower adherence to anticancer treatment, prolonged hospitalization, and higher mortality.^29^ Adherence to antidepressant treatment has been shown to have a positive impact on mortality in patients with depression and cancer.^30^ It is possible that the prescription of antidepressants may act as a mediator, indirectly influencing the overall mortality risk in patients with HCC by mitigating the negative health effects of depression.

In addition to observing lower risk of overall mortality, we observed that antidepressant use after HCC diagnosis was inversely associated with cancer-specific mortality. This association remained consistent when specifically analyzing different antidepressants including SSRIs, SNRIs, and TCAs. This might be explained by previously reported apoptotic effects of serotonergic antidepressants on cancer through the regulation of growth stimulatory 5-HT activity connected to biochemical pathways involving mitogen-activated protein kinase, mitochondrial membrane potential, extracellular signal-regulated kinases, protein kinase B, and nuclear transcription factor-κB.^31^ The apoptotic activity of TCAs and SSRIs has been observed in different tumor cell lines including HCC.^8,10,11,32,33^ In addition, some SSRIs (fluoxetine and sertraline) have been reported to be effective chemosensitizers that increase the cytotoxicity of anticancer drugs and suppress the growth of HCC cells.^32,34^ However, our results did not reveal additional beneficial associations when examining the interaction between postdiagnosis antidepressant use and HCC treatments, including chemotherapy and sorafenib. The lack of synergistic interaction may be attributed to the different mechanisms of action between antidepressants and chemotherapy or sorafenib. Further research is warranted to explore the potential biological mechanisms underlying the inverse association between antidepressants and HCC mortality.

Despite biological plausibility, studies have presented mixed results for associations of antidepressants with mortality in patients with cancer. Some studies have reported that antidepressant use in patients with depression and cancer has a beneficial effect on premature mortality,^30^ and antidepressant use was found to be associated with reduced mortality in lung cancer specifically.^35^ However, other researchers have reported contradictory findings, for example, that use of antidepressants in patients with melanoma, breast, prostate, lung, colorectal, and other cancers is associated with increased mortality.^21,36^

### Strengths and Limitations

This study has several strengths. By using a nationally registered data set with a 99% coverage of the Taiwanese population, this large-scale cohort study has national representativeness and low potential for selection bias. Furthermore, we adjusted for a range of potential confounders and took into consideration the potential induction periods of antidepressants, the timing of antidepressant use (before and after HCC diagnosis), and types of mortality (overall and cancer specific). In our analysis of antidepressant use after HCC diagnosis, we used time-varying exposure to minimize immortal time bias. Therefore, this study yielded robust results on the association between mortality and postdiagnosis antidepressant use in patients with HCC.

Several limitations may affect the results and interpretations of this study. First, information on some potential confounders was not available in the NHIRD, including adherence to antidepressant use, smoking status, body mass index, nutritional status, other health-related factors, and laboratory test results, and we thus could not include and adjust for them in our statistical models. Second, our study may have misclassification bias because some participants may not have adhered to antidepressant medication. However, such misclassification usually results in an underestimation of the effect of interest^37^; therefore, the true association may be more pronounced. Third, the low cancer-specific mortality in our study could be attributed to our definition based on the primary cause of death certificate records, where complications of HCC (such as liver cirrhosis or infectious complications) may appear as primary causes of death, leading to an underestimation of cancer-specific mortality. Fourth, this study used Taiwanese population data, and the results might not be generalizable to other countries. Finally, it is important to acknowledge that the current evidence supporting the use of antidepressants in HCC is limited, and there are no HCC management consensus guidelines recommending their use.^38,39^ Given that antidepressant use in our study was not specifically targeted at HCC treatment and the study design was retrospective and observational, caution is warranted when interpreting the observed associations.

## Conclusions

In this large population-based HCC cohort study, antidepressant use after HCC diagnosis was associated with lower overall and cancer-specific mortality among patients with HCC. Our study provides promising empirical results indicating that antidepressants may have utility as anticancer therapeutics in patients with HCC. However, our findings should be interpreted cautiously because the associations found in this observational study may not indicate causality and may be affected by residual confounding or biases. Definitive evidence would require evaluation in randomized clinical trials.

## Notes

Supplement 1. **eTable 1.** Association Between Postdiagnosis Antidepressant Use and Mortality in Patients With HCC Using Different Induction Periods **eTable 2.** Interaction Between Antidepressant Use and Chemotherapy/Sorafenib for Mortality Risk in Patients With HCC

Supplement 2.
Data Sharing Statement
