# Supplementary material for: Antidepressant Use and Mortality Among Patients With Hepatocellular Carcinoma
Source: JAMA Netw Open. 2023 Sep 6;6(9):e2332579. doi: 10.1001/jamanetworkopen.2023.32579 (PMC10483320; doi:10.1001/jamanetworkopen.2023.32579)
Supplement: Supplement 1. — eTable 1. Association Between Postdiagnosis Antidepressant Use and Mortality in Patients With HCC Using Different Induction Periods eTable 2. Interaction Between Antidepressant Use and Chemotherapy/Sorafenib for Mortality Risk in Patients With HCC [file jamanetwopen-e2332579-s001.pdf]

Supplemental Online Content

Huang K, Chen Y, Stewart R, Chen VCH. Antidepressant use and mortality among patients with hepatocellular carcinoma. *JAMA Netw Open*. 2023;6(9):e2332579. doi:10.1001/jamanetworkopen.2023.32579

eTable 1. Association between postdiagnosis antidepressant use and mortality in patients with HCC using different induction periods
eTable 2. Interaction between Antidepressant Use and Chemotherapy/Sorafenib for Mortality Risk in Patients with HCC

This supplemental material has been provided by the authors to give readers additional information about their work.

© 2023 Huang K et al. *JAMA Network Open.*

eTable 1 Association between postdiagnosis antidepressant use and mortality in patients with HCC using different induction periods

	Overall mortality		Cancer-specific mortality	
	0-day induction period<br>Adjusted HR (95% CI)	180-day induction period<br>Adjusted HR (95% CI)	0-day induction period<br>Adjusted HR (95% CI)	180-day induction period<br>Adjusted HR (95% CI)
**Antidepressants**	0.78 (0.78-0.79)	0.64 (0.63-0.65)	0.73 (0.68-0.78)	0.58 (0.53-0.62)
**SSRI**	0.68 (0.66-0.69)	0.59 (0.58-0.61)	0.60 (0.53-0.68)	0.51 (0.44-0.58)
**SNRI**	0.65 (0.62-0.68)	0.56 (0.54-0.59)	0.58 (0.46-0.72)	0.50 (0.39-0.64)
**TCA**	0.65 (0.64-0.66)	0.56 (0.55-0.57)	0.59 (0.54-0.64)	0.50 (0.45-0.55)

Abbreviations: HR, hazard ratio; HCC, hepatocellular carcinoma; SSRI, selective serotonin reuptake inhibitor; SNRI, serotonin-norepinephrine reuptake inhibitor; TCA, tricyclic antidepressant

Adjusted for age, sex, low-income, prediagnostic comorbidities (HBV, HCV, liver cirrhosis, alcohol use disorder), CCI, and HCC treatment (operation, RFA, TAE/TACE, radiotherapy, chemotherapy, sorafenib)

Non-user were defined as HCC patients without an antidepressant prescription in the 1 year before and after the HCC diagnosis

© 2023 Huang K et al. *JAMA Network Open.*

eTable 2 Interaction between Antidepressant Use and Chemotherapy/Sorafenib for Mortality Risk in Patients with HCC

	Antidepressant * Chemotherapy Adjusted HR (95% CI)[a]	Antidepressant * Sorafenib Adjusted HR (95% CI)[b]
**Overall mortality**		
Antidepressants	1.41 (1.34-1.48)	1.61 (1.54-1.68)
SSRI	1.26 (1.15-1.37)	1.70 (1.57-1.84)
SNRI	1.49 (1.27-1.73)	2.11 (1.85-2.41)
TCA	1.50 (1.41-1.59)	1.67 (1.58-1.77)
**Cancer-mortality**		
Antidepressants	1.71 (1.22-2.39)	2.03 (1.71-2.41)
SSRI	1.37 (0.72-2.63)	2.67 (1.99-3.58)
SNRI	2.85 (1.29-6.31)	3.55 (2.17-5.80)
TCA	1.70 (1.11-2.58)	2.21 (1.80-2.72)

Abbreviations: HR, hazard ratio; HCC, hepatocellular carcinoma

[a] Adjusted for age, sex, low-income, prediagnostic comorbidities (HBV, HCV, liver cirrhosis, alcohol use disorder), CCI, and HCC treatment (operation, RFA, TAE/TACE, Radiotherapy, sorafenib)

[b] Adjusted for age, sex, low-income, prediagnostic comorbidities (HBV, HCV, liver cirrhosis, alcohol use disorder), CCI, and HCC treatment (operation, RFA, TAE/TACE, Radiotherapy, Chemotherapy)

© 2023 Huang K et al. *JAMA Network Open.*
