# Supplementary material for: Antidepressant Use and Mortality Among Patients With Hepatocellular Carcinoma
Source: JAMA Netw Open. 2023 Sep 6;6(9):e2332579. doi: 10.1001/jamanetworkopen.2023.32579 (PMC10483320; doi:10.1001/jamanetworkopen.2023.32579)
Supplement: Supplement 2. — Data Sharing Statement [file jamanetwopen-e2332579-s002.pdf]

Data Sharing Statement

Huang. Antidepressant Use and Mortality Among Patients With Hepatocellular Carcinoma. *JAMA Netw Open*. Published September 06, 2023. doi:10.1001/jamanetworkopen.2023.32579

Data
Data available: No

Additional Information
Explanation for why data not available: Data supporting the results of this study are available from the Ministry of Health and Welfare of Taiwan, but there are restrictions on the availability of these data, which were used under license in this study and are therefore not publicly available. However, these data are available to the authors upon reasonable request and with permission from the Ministry of Health and Welfare of Taiwan.
